# Authors’ rebuttal to Integrated Risk Information System (IRIS) response to “Assessing risk of bias in human environmental epidemiology studies using three tools: different conclusions from different tools”

**DOI:** 10.1186/s13643-022-01894-8

**Published:** 2022-03-23

**Authors:** Stephanie M. Eick, Dana E. Goin, Juleen Lam, Tracey J. Woodruff, Nicholas Chartres

**Affiliations:** 1 Gangarosa Department of Environmental Health, Rollins School of Public Health, Emory University, Atlanta, GA USA; 2 Program on Reproductive Health and the Environment, Department of Obstetrics, Gynecology and Reproductive Sciences, University of California, San Francisco, San Francisco, CA USA; 3 Department of Public Health, California State University, East Bay, Hayward, CA USA

**Keywords:** Risk of bias, Systematic review, Risk assessment

## Abstract

This letter responds to the US Environmental Protection Agency’s Integrated Risk Information System (IRIS) program letter by Radke et al. (2021) that was published in response to the application of the IRIS risk of bias tool in our recent study “Assessing risk of bias in human environmental epidemiology studies using three tools: different conclusions from different tools.” Their letter stated that we misrepresented the IRIS approach. Here, we respond to their three points raised and how we did not misrepresent their tool and also identified areas for improvement: (1) why it should be expected that different reviewers could reach different conclusions with the IRIS tool, as ratings are subject to reviewer judgment; (2) why our interpretation that “low” or “uninformative” studies could be excluded from a body of evidence was reasonable; and (3) why we believe the use of a rating system that generates an overall rating based on an individual domain or a combination of identified deficiencies essentially acts as a score and assumes that we know empirically how much each risk of bias domain should contribute to the overall rating for that study. We have elaborated on these points in our letter.

We appreciate the opportunity to respond to comments raised by the Integrated Risk Information System (IRIS) program letter by Radke et al. (2021) [1]. We acknowledge that the IRIS program is critically important to the US Environmental Protection Agency (EPA)’s mission of protecting human health, and the “*ORD staff handbook for developing IRIS assessments*” (hereafter referred to as the “Handbook”) is another important milestone in the program’s adoption of systematic review methods [2]. We commend the IRIS program on the important progress it has made in adopting and implementing systematic review methods in conducting IRIS assessments. Although we identified the need to refine this risk of bias (ROB) approach in our study, the Handbook provides a strong foundation for conducting IRIS assessments and should serve as the basis for planning and conducting systematic reviews across all of EPA’s programs. Further, the National Academies of Sciences, Engineering, and Medicine have recently published a report on the Hanbook, *Review of U.S. EPA's ORD Staff Handbook for Developing IRIS Assessments: 2020 Version (2021) (cite National Academies of Sciences, Engineering, and Medicine 2021. Review of U.S. EPA's ORD Staff Handbook for Developing IRIS Assessments: 2020 Version. Washington, DC: The National Academies Press.* 10.17226/26289.) that also acknowledged this progress but also found that the Handbook and IRIS assessments could be improved in several areas, including the ROB approach.

The IRIS program letter was in response to the application of the IRIS ROB tool in our recent study “*Assessing risk of bias in human environmental epidemiology studies using three tools: different conclusions from different tools.*” [3]. Their letter stated that we were wrong about the application of their tool and outlines three clarifications regarding the application of the IRIS tool to which we respond below.

## Clarification one

First, Radke et al. (2021) state that they “did not reach the same conclusions with our [PRHE] analysis of the same studies” [1] when comparing their study ratings to the ratings that we reported in our original study, and that the study evaluation process of a systematic review should first begin “with development and pilot testing exposure-outcome specific criteria that identify the information and appropriate methods needed to apply the evaluation ratings in each domain. These criteria are based on the state of knowledge about the toxicokinetics of the chemical being assessed, exposure assessment methods, and the epidemiological standard of practice for specific outcomes.” [1].

We agree with Radke et al. (2021) that these steps are necessary to ensure transparency and consistency of systematic reviews. We also agree that this step is needed to appropriately evaluate the ratings for each domain. However, in their response [1], Radke et al. (2021) incorrectly state that these methods were not part of our study. In actuality, we conducted pilot testing where reviewers, which we described in our study “… independently rate[d] [risk of bias] ROB for one article…. The two reviewers reviewed the first study and then met, compared ratings, discussed discrepancies, came to consensus on ratings, and standardized their approach.” [3]. As part of this process, the two reviewers modified the questions to be applicable to the exposure-outcome relationship we were testing, PBDEs (polybrominated diphenyl ethers) and IQ (intelligence quotient). Prior to the pilot testing “...the two reviewers (SME, DEG) completed training (approximately 4 h) on assessing ROB in epidemiology studies with a systematic review expert (JL). Trainings included a broad overview on assessing ROB and specific clarification on the application of each tool.” [3].

This training was conducted by the lead author of the systematic review on PBDE and IQ [4] that was part of our study (JL) and as part of that training specific details regarding the interpretation of relevant exposures and outcomes decided on by the collection of authors on that systematic review were shared to inform the applications of additional tools. We also recognize in the limitations of our study that “Our team did not include a neurodevelopment or biomarker assessment subject matter expert, and inclusion of these individuals may have led to different ratings for some of the domains.” [3].

The overall study confidence ratings of the IRIS tool are subject to reviewer judgment and expert opinion, as we note in our study [3]. Thus, it is expected that different groups with differing levels of expertise and resources would reach different conclusions. This is the very reason why we and other authoritative bodies including the NASEM recommend to *not* use an overall study confidence rating to evaluate the body of evidence [5]. As such, the IRIS program letter by Radke et al. (2021) demonstrates the essential problem with including an overall study rating, which may lead to the exclusion of a study or could inappropriately downgrade study findings in the overall assessment.

## Clarification two

Second, Radke et al. (2021) state that our assertion that studies deemed “low confidence” or “uninformative” overall would be removed from the overall body of evidence is not consistent with the IRIS Handbook. In Radke et al. (2021), their rationale is that the IRIS Handbook states that “Low confidence results are given less weight compared to high or medium confidence results during evidence synthesis and integration” [1]. Furthermore, they state that “Low confidence studies are included in the evidence synthesis, and comparisons of these results with those of high or medium confidence studies facilitate the review of consistency (i.e., between study heterogeneity).” [1]. However, the authors do not include the entirety of the text from the IRIS Handbook to support their response. The IRIS Handbook states that “Low confidence results are given less weight compared to high or medium confidence results during evidence synthesis and integration, and are generally not used as the primary sources of information for hazard identification or derivation of toxicity values unless they are the only studies available.” [2].

A reasonable interpretation of the entire sentence is that if “low confidence” studies are “generally not used as the primary sources of information for hazard identification,” this would indicate that they should be excluded from consideration. We noted this in our manuscript as a potential limitation “...this may reduce the available evidence to assess the harms of environmental exposures by erroneously excluding studies, which leads to inaccurate conclusions about the quality of the body of evidence.” [3].

Radke et al. (2021) further state that “Uninformative studies, on the other hand, are excluded from further evidence synthesis, consistent with the practices of NTP RoC [6] and ROBINS-I [7], because the evaluation found “serious flaw(s) [that] make the study results unusable for informing hazard identification” [2]. Our concerns with this approach were highlighted in the NASEM report on the Handbook (cite National Academies of Sciences, Engineering, and Medicine 2021. Review of U.S. EPA's ORD Staff Handbook for Developing IRIS Assessments: 2020 Version. Washington, DC: The National Academies Press. 10.17226/26289.), where it was stated that " EPA provided data from recent IRIS assessments showing that the proportion of human studies rated as “uninformative” and excluded from further consideration ranged from 0 to 50 percent, and 0 to 41.5 percent for animal studies. Thus, depending on the IRIS assessment, excluding studies at the study evaluation stage could lead to a substantial proportion of excluded studies due to a critically deficient rating in one domain." The NASEM reccommended "The handbook should not use the results of study evaluation as eligibility criteria for the systematic review". Our comments are also consistent with the 2021 NASEM report which evaluated the US EPA’s Toxic Substances Control Act (TSCA) systematic review methodology that recommended “Do not exclude studies based on risk of bias, study quality, or reporting quality” [5] and stated “While there is inevitably variation in the internal validity and risk of bias across individual studies, it is standard practice to include all studies, even the studies with a high risk of bias into the evidence synthesis. The most appropriate method to exclude studies from evidence synthesis is based on predefined exclusion criteria that should preclude an irrelevant study from being evaluated…. Once a study is determined to be eligible, the study could be included in the synthesis and the risk-of-bias assessment and its limitations accounted for in any qualitative or quantitative synthesis…..In the synthesis step, low-quality studies may be excluded as a sensitivity analysis, but it is inappropriate to leave them out of synthesis completely.” [5] (emphasis added).

## Clarification three

Third, Radke et al. (2021) state that we object to the use of an overall study rating, and state that “There is explicitly not a weighting of domains or quantitative scheme for reaching these overall ratings; one impactful limitation or a combination of identified deficiencies can result in a rating of low confidence.” [1]. We agree with this and we highlighted this in our study “an important distinction between the IRIS, OHAT, and TSCA tools is that the IRIS tool includes a subjective indicator, as opposed to a weighted average or similar, for overall study quality.” [3].

However, although the Handbook’s ROB evaluation does not explicitly use quantitative scores or a weighted average, the use of a rating system that generates an overall rating based on an individual domain “or a combination of identified deficiencies” [2] essentially acts as a score and assumes that we know empirically how much each ROB domain should contribute to the overall rating for that study. For example, if a study is rated as “deficient” in the domains of “exposure assessment” and “selection of participants” and combined it makes the whole study “low confidence,” this is essentially equivalent to adding a weight, quantitative or not, to determine that such limitations outweigh how well a study was conducted in the other domains, which then leads to the generation of the overall study rating. However, the use of ‘quality scores’ has not been able to distinguish between studies with a high and low ROB in meta-analyses and empirical evidence is lacking to establish how each ROB item should be weighted [6, 7].

There is empirical evidence that inadequate application of randomization and blinding results in overestimation of efficacy of drug effects [8, 9]. However, such empirical examinations of the association between the methods and results for each ROB domain in the ROBINS-I, and the Handbook’s subsequent adaptation of ROBINS-I, have not been conducted and it is unclear whether these tools would stand up to such an empirical assessment. Therefore, to rate a study as overall “low” or “medium” confidence based on measures not validated is concerning and would likely result in exclusion of studies that are informative to the risk assessment.

Radke et al. (2021) go on to state that “The analysis by Eick et al. acknowledges that there is flexibility in the application of the overall study confidence rating, but incorrectly presents it as an override of what they interpret as a more deterministic approach where the number of good, adequate, and deficient ratings are counted to obtain an overall rating.” [1] We in fact do not do this and simply presented the results as we interpreted the instructions, and in an effort to present a balanced and rigorous representation of the results, conducted an additional sensitivity analysis “while we used the instructions explicitly to rate the ROB, in an effort to examine the robustness of our original findings, we also conducted a sensitivity analysis to determine if the overall study confidence rating would vary with alternative guidance.” [3].

Our sensitivity analysis highlights why the Handbook is ambiguous and prone to excluding studies without scientific justification “For example, the IRIS tool allows studies to be classified as “medium” study confidence if there is a deficient rating in a domain that is considered to have less influence on the direction of the effect estimate. However, the handbook does not define which domains have less influence and we were unable to find scientific evidence to support judgments of certain domains as being more influential than others.” [3].

Finally, we agree that “the IRIS study evaluation approach is a transparent method to inform certainty” in evidence synthesis decisions made in IRIS assessments based on how the current IRIS program scientists have applied this tool [1]. Our study did not at any stage suggest that approach lacked transparency. However, as we highlighted in our study, the current instructions in the IRIS Handbook can be interpreted in different ways when determining whether a study is considered overall “low”, “moderate,” or “high” confidence, and therefore, we do not believe it will “ensure consistency in the development of IRIS health assessments” unless there is the same group of review authors agreeing on what domains they consider to be more important as a ROB when determining the overall study confidence [1].

## Data Availability

All data generated or analyzed during this study are included in this published article [and its supplementary information files].

## Abbreviations

IRIS: Integrated Risk Information System
PBDEs: Polybrominated diphenyl ethers
IQ: Intelligence quotient
ROB: Risk of bias
EPA: Environmental Protection Agency
