# Correction: Authors’ rebuttal to Integrated Risk Information System (IRIS) response to “Assessing risk of bias in human environmental epidemiology studies using three tools: different conclusions from different tools”

**DOI:** 10.1186/s13643-022-01967-8

**Published:** 2022-05-05

**Authors:** Stephanie M. Eick, Dana E. Goin, Juleen Lam, Tracey J. Woodruff, Nicholas Chartres

**Affiliations:** 1 Gangarosa Department of Environmental Health, Rollins School of Public Health, Emory University, Atlanta, GA USA; 2 Program on Reproductive Health and the Environment, Department of Obstetrics, Gynecology and Reproductive Sciences, University of California, San Francisco, San Francisco, CA USA; 3 Department of Public Health, California State University, East Bay, Hayward, CA USA

**Correction to: Syst Rev 11, 53 (2022)**

10.1186/s13643-022-01894-8

In the original publication of this article [1], the new reference citation has been included in the body text repeatedly and not added as an endnote.

Further, the National Academies of Sciences, Engineering, and Medicine have recently published a report on the Hanbook, Review of U.S. EPA’s ORD Staff Handbook for Developing IRIS Assessments: 2020 Version (2021) (cite National Academies of Sciences, Engineering, and Medicine 2021. Review of U.S.EPA’s ORD Staff Handbook for Developing IRIS Assessments: 2020 Version. Washington, DC: The National Academies Press. 10.17226/26289) that also acknowledged this progress but also found that the Handbook and IRIS assessments could be improved in several areas, including the ROB approach.

Our concerns with this approach were highlighted in the NASEM report on the Handbook (cite National Academies of Sciences, Engineering, and Medicine 2021. Review of U.S.EPA’s ORD Staff Handbook for Developing IRIS Assessments: 2020 Version. Washington, DC: The National Academies Press. 10.17226/26289), where it was stated that " EPA provided data from recent IRIS assessments showing that the proportion of human studies rated as “uninformative” and excluded from further consideration ranged from 0 to 50 percent, and 0 to 41.5 percent for animal studies.

The corrected citations are shown below and the original article has been corrected.

Further, the National Academies of Sciences, Engineering, and Medicine [NASEM] have recently published a report on the Handbook, *Review of U.S. EPA’s ORD Staff Handbook for Developing IRIS Assessments: 2020 Version* [3] that also acknowledged this progress but also found that the Handbook and IRIS assessments could be improved in several areas, including the ROB approach.

Our concerns with this approach were highlighted in the NASEM report on the Handbook [3], where it was stated that "EPA provided data from recent IRIS assessments showing that the proportion of human studies rated as “uninformative” and excluded from further consideration ranged from 0 to 50 percent, and 0 to 41.5 percent for animal studies. Thus, depending on the IRIS assessment, excluding studies at the study evaluation stage could lead to a substantial proportion of excluded studies due to a critically deficient rating in one domain."
